# The Use of Synthetic Mesh in Reconstructive, Revision, and Cosmetic Breast Surgery

**DOI:** 10.1007/s00266-013-0171-8

**Published:** 2013-07-17

**Authors:** Hilton Becker, Jeffrey G. Lind

**Affiliations:** 1 Hilton Becker Clinic of Plastic Surgery, 670 Glades Road #220, Boca Raton, FL 33431 USA; 2 Charles E. Schmidt College of Medicine, Florida Atlantic University, Boca Raton, FL 33431 USA; 3 Department of Plastic and Reconstructive Surgery, Cleveland Clinic Florida, 2950 Cleveland Clinic Blvd, Weston, FL 33331 USA

**Keywords:** Absorbable synthetic surgical mesh, TIGR^®^ Matrix Surgical Mesh, Long-term resorbable surgical mesh, Acellular dermal matrix, Reconstructive breast surgery, Tissue expander implant breast surgery

## Abstract

**Background:**

Recent evidence suggests that the use of acellular dermal matrices in prosthetic breast reconstruction, revision, or augmentation may be associated with an increased risk of complications. In this article we report our results of a potential alternative, using a new long-term resorbable synthetic matrix in these cases.

**Methods:**

A retrospective study was performed evaluating 11 primary breast reconstructions (19 breasts), 43 secondary reconstructions (77 breasts), 3 augmentation/augmentation mastopexys (6 breasts), and 5 mastopexys (10 breasts) in 62 patients using TIGR^®^ Matrix Surgical Mesh.

**Results:**

Follow-up ranged from 9.4 to 26.1 months with an average follow-up of 16.5 months. Average age was 54 years. The number of patients who had prior radiation was 9 (14.5 %). Four patients (6.5 %) were smokers. Postoperative breast complications included necrosis of two flaps (1.8 %), two seromas requiring drainage (1.8 %), four infection/extrusions (3.6 %), two relapses of inframammary fold/malposition (1.8 %), and two with rippling (1.8 %). Other complications included six cases of asymmetry that required a corrective procedure. In a variety of breast surgery cases very good aesthetic results were achieved.

**Conclusion:**

The long-term absorbable synthetic matrix, TIGR^®^ Matrix Surgical Mesh, shows potential when used as temporary reinforcement in patients undergoing breast reconstruction or breast surgery revisions and in primary aesthetic procedures, and it appears to be a viable alternative to the use of acellular dermal matrices.

**Level of Evidence IV:**

This journal requires that authors assign a level of evidence to each article. For a full description of these Evidence-Based Medicine ratings, please refer to the Table of Contents or the online Instructions to Authors http://www.springer.com/00266.

## Introduction

The use of tissue expanders has become the most common technique of performing breast reconstruction as evidenced by the 2011 ASPS Plastic Surgery Statistics Report [2]. Chedomir Radovan, MD, a great innovator of the 1970 s, is credited with the development of the Radovan breast expander [31, 32]. He initially described placing tissue expanders in the subcutaneous position. This technique evolved over time leading to the placement of the expander in a submuscular position. This approach to expander-based breast reconstruction is used to maintain total muscle coverage in order to protect the expander from the incision and potential exposure [3]. This technique includes elevating the pectoralis major muscle, serratus anterior muscle, and the anterior fascia of the rectus abdominus muscle [4, 7]. The rigidity of the fascia inferiorly restricts inferior pole expansion and often results in a high-riding implant. Furthermore, with complete muscle coverage the inframammary fold is ill-defined [29, 30]. Complete muscle coverage was necessary when a more extensive mastectomy was performed in order to protect the implant from exposure beneath the large incision.

The advent of the skin-sparing mastectomy enabled the muscle to be detached inferiorly where the lower skin flap affords coverage to the implant. Although expansion is facilitated with the release of the muscle inferiorly [34], pectoral muscle retraction and bottoming out of the implant became problems [22]. Therefore, the inferior edge of the muscle was sutured to the fascia. However, sutures alone were often ineffective in holding the muscle in position. The tension often resulted in disruption as the sutures cut through the tissues. Acellular dermal matrices (ADM) offered a viable solution to this problem [9, 33, 35, 37]. The ADM reinforces the muscle and also provides supplemental tissue to the space between the released muscle and the inframammary fold. It allows the pectoralis muscle to be released, expands the space, allows fixation of the inframammary fold, and fills in the tissue void between the inferior edge of the pectoralis muscle and the inframammary fold. However, due to recently reported problems encountered with ADM, including seroma, infection, slow vascularization, disruption, reconstructive failure, patient concerns, and costs, we looked at a new long-term resorbable synthetic mesh as a potential alternative [10, 13, 17, 19, 40–43]. Surgical mesh has multiple fixation points thus offering greater tissue fixation compared to sutures. This mesh also functions as a scaffold facilitating native tissue in-growth.

Initially, permanent synthetic meshes (PTFE and Ultrapro) were used but they proved to be too rigid. We then resorted to absorbable mesh (Vicryl), which functioned well for the first few months, but rapid absorption resulted in bottoming out in certain cases. We therefore began using a new synthetic long-term absorbable scaffold known as TIGR^®^ Matrix Surgical Mesh (Novus Scientific Pte Ltd, Singapore). Here we report on the use of this new long-term resorbable synthetic mesh (TIGR^®^ Matrix) in over 50 breast cases. The categories of breast surgery in this study include primary reconstruction, reconstruction revision, augmentation/mastopexy revision, augmentation/augmentation mastopexy, and mastopexy. The aim of this study was not to compare TIGR^®^ Matrix to ADMs or other similar products but to look at this mesh as a potential alternative to ADMs in a variety of breast surgery cases. We hypothesize that TIGR^®^ Matrix is a viable alternative to ADMs.

## Materials and Methods

A retrospective review was performed on patients who underwent a primary or secondary breast reconstructive surgical procedure as well as patients who underwent a primary aesthetic procedure or aesthetic surgery revision that included the use of TIGR^®^ Matrix Surgical Mesh from 2011 to 2012. All patients meeting these criteria were included. There were no exclusion criteria. All cases were performed by a single surgeon in a private practice setting. Informed verbal and written consent were obtained in all cases. The principles outlined in the Declaration of Helsinki were strictly followed. The smooth Spectrum adjustable implant [5, 6, 8] or a smooth silicone gel implant was used in all cases. Smooth implants were used because our experience does not show an increased frequency of capsular contracture associated with their use. Also, they are easier to work with and have been shown in our experience to have a lower postoperative seroma rate. The adjustable implants were used in all immediate reconstructions where there was a concern about circulation, in patients who had prior radiation, and in cases of excessive scarring. In patients undergoing revision surgery where capsules were present, either capsulectomy or aggressive capsular scoring was performed in order to facilitate vascularization of the mesh. Regular clinical follow-up was done in all patients. Small biopsies of the mesh were taken for histological analysis in patients who had to return to the operating room.

Outpatient charts from this single private practice were reviewed retrospectively. Data collected included patient age, follow-up time, history of prior radiation, history of smoking, reasons for surgery, procedures performed, perioperative findings, postoperative complications, and need for postoperative interventions.

TIGR^®^ Matrix Surgical Mesh is a macroporous mesh knitted from two different degradable fibers: a fast-degrading fiber and a slow-degrading fiber. The fast-degrading fiber is a copolymer between glycolide and trimethylene carbonate, and the slow-degrading fiber is mainly a copolymer between lactide and trimethylene carbonate. The materials used in TIGR^®^ Matrix are all very well known from the suture industry and have been used clinically since 1970 in a vast number of different medical devices [27]. Both fibers degrade into small molecules that are readily absorbed and excreted from the body [20, 28, 39]. The fast-degrading fiber gives extra support during the first wound-healing phase and is totally resorbed within 4 months after implantation. About 2 weeks after implantation TIGR^®^ Matrix will gradually become softer and more flexible due to the ongoing degradation in the fast-degrading fiber, unlocking the knitting pattern of the slow-degrading fiber. The slow-degrading fibers keep their mechanics up to 6–9 months and are completely resorbed after ~3 years due to degradation by hydrolysis [18].

## Results

A total of 62 patients (112 breasts) composed the study population. The mean age of the patients at the time of operation was 54 years. Average follow-up was 16.5 months (range 9.4–26.1). No patients were lost to follow-up. Nine patients (14.5 %) had had prior radiation and four patients (6.5 %) were active smokers (Table 1).

**Table 1:** Patient characteristics

Average follow-up (months) [min-max]	16.5 [9.4–26.1]
Average age (years)	54
No. of patients with prior radiation	9 (14.5 %)
No. of smokers	4 (6.5 %)

Reasons for surgery included capsular contraction, elevation or reconstruction of the inframammary fold, implant removal or replacement, asymmetric primary results, scar revision, and implant repositioning. A total of 11 primary reconstructions (19 breasts), 43 secondary reconstructions (77 breasts), three augmentation/augmentation mastopexys (six breasts), and five mastopexys (10 breasts) were performed using TIGR^®^ Matrix Surgical Mesh. Of the 43 secondary reconstructions, 30 were reconstruction revisions and 13 were augmentation/mastopexy revisions (Table 2).

**Table 2:** Procedures performed

Procedure	No. of patients	No. of breasts
Primary reconstruction	11	19
Reconstruction revision	30	51
Augmentation/mastopexy revision	13	26
Augmentation/augmentation mastopexy	3	6
Mastopexy	5	10
Total	62	112

Perioperative findings included two failed ADM grafts (two patients) as they were fragmented or not integrated at all. Both of these patients had received prior radiation. The biologic material implanted in their primary reconstruction performed at different centers was found to be nonintegrated and ineffective. The ADM grafts were thus removed and replaced with TIGR^®^ Matrix. There were no intraoperative complications.

**Fig. 1:**
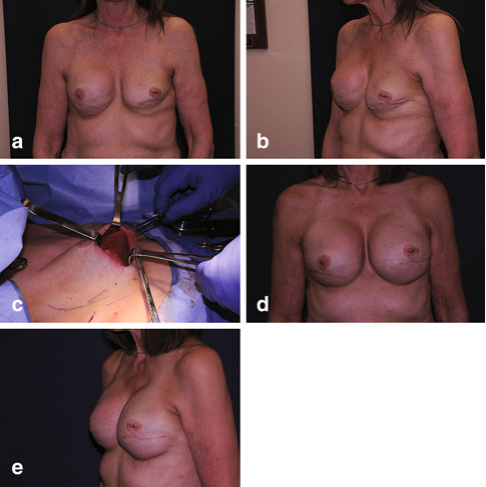
**a** Preoperative photo of patient following bilateral breast reconstruction with bilateral scar adhesions present. **b** Preoperative lateral view. **c** Bilateral open capsulotomies performed, TIGR^®^ mesh support placed, silicone gel implants inserted. **d** Final result. **e** Final result, lateral view

**Fig. 2:**
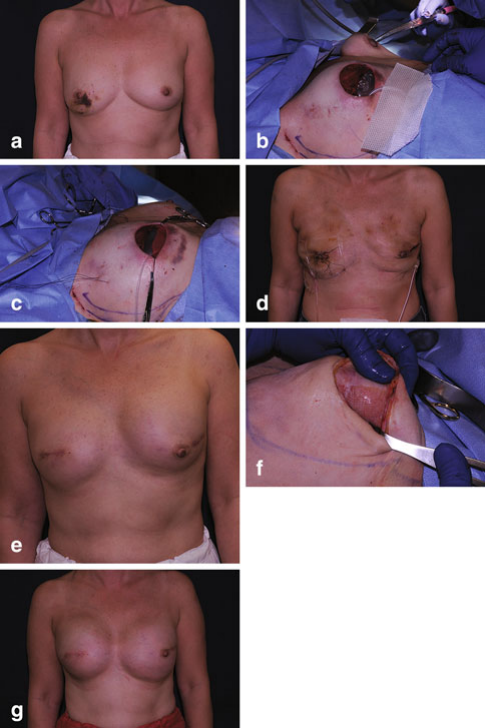
**a** Preoperative photo of patient with right breast carcinoma. **b** Insertion of temporary expander to assess submuscular pocket. **c** Expander in position and TIGR^®^ mesh sutured to inferior edge of muscle. **d** Immediate postoperative result. **e** Following expansion. **f** Open capsulotomy performed prior to insertion of gel implant at 4 months; note well incorporated mesh. **g** Final result

**Fig. 3:**
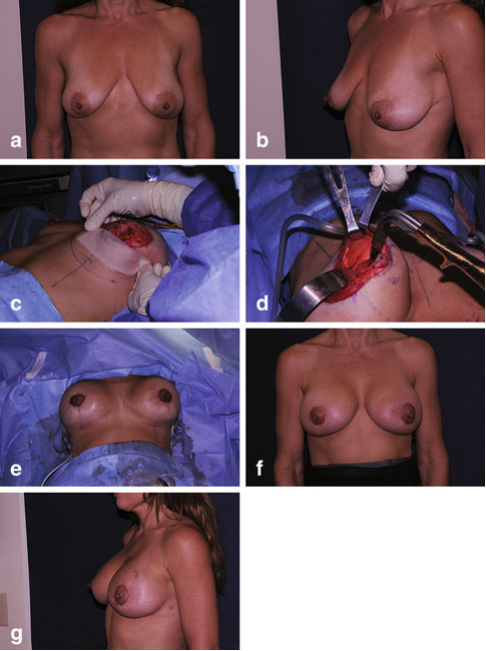
**a** Preoperative photo of patient with bilateral breast ptosis. **b** Preoperative lateral view. **c** Following subareola mastopexy, mesh is prepared for insertion as a hammock. **d** Mesh sutured into position. **e** Skin closure; note elevation of breasts. **f** Final result. **g** Final result, lateral view

Very good cosmetic results were obtained in a variety of breast surgery cases (Figs. 1, 2, 3 4).

**Fig. 4:**
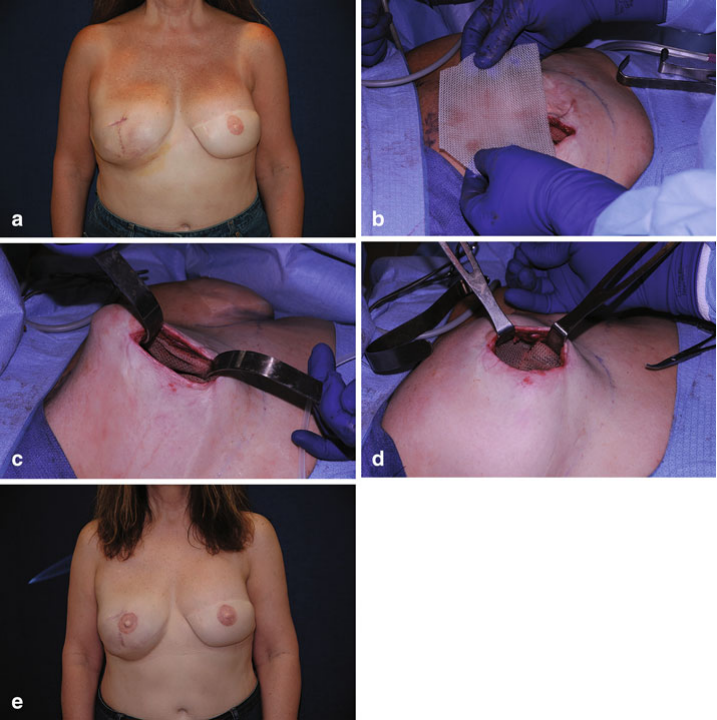
**a** Preoperative photo of patient following bilateral breast reconstruction with expander reconstruction of the right breast. **b** Revision of the right breast with insertion of TIGR^®^ mesh support and insertion of silicone gel breast implant. **c** Mesh sutured into position. **d** Closure of mesh over implant. **e** Final result

**Fig. 5:**
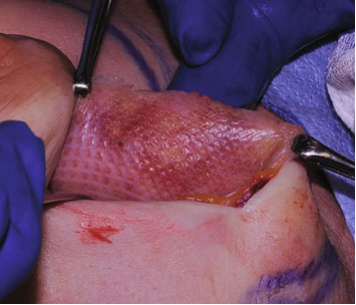
TIGR^®^ mesh at 4 months. Note the fibers are well incorporated with collagen laid down over the mesh

**Fig. 6:**
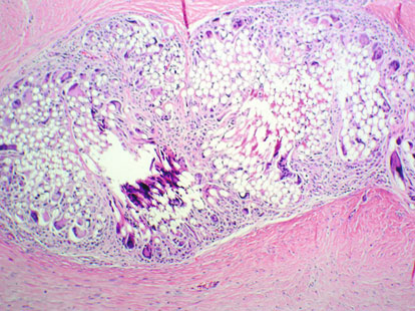
Histology at 5 months. The mesh fibers are still present surrounded by a layer of fibroblasts and collagen

The total postoperative complication rate was 20.5 % of breasts. The total postoperative complication rate of patients requiring an intervention was 15.2 % of breasts. Postoperative breast complications included necrosis in two flaps (1.8 %), two seromas (1.8 %), four infection/extrusions (3.6 %), two relapses of inframammary fold/malposition (1.8 %), and two with rippling (1.8 %) (Table 3). Table 4 gives the list of operations and respective complications. All but one patient required an intervention (the one with mild rippling did not need an intervention). Another complication requiring intervention was asymmetry in six cases (5.4 %). Grossly, the mesh was well incorporated with deposited collagen in all patients who had to return to the operating room (Fig. 5). Histological analysis of the incorporated TIGR^®^ Matrix showed the mesh to still be present with surrounding fibroblasts and collagen deposition (Fig. 6).

**Table 3:** Complications

Complication	No. of breasts with complications	% (of total breasts)	No. of breasts requiring intervention
Flap necrosis	2	1.8	2
Seroma	2	1.8	2
Infection/extrusion	4	3.6	4
Relapse of IMF/malposition	2	1.8	2
Rippling	2	1.8	1
Asymmetry	11	9.8	6
Total	23	20.5 %	17 (15.2 % of total breasts)

**Table 4:** Operation with associated complications

Surgery	No. of breasts	Flap necrosis	Seroma	Infection/extrusion	Relapse of IMF	Rippling	Asymmetry requiring revision
Primary reconstruction	19	1	2	3	0	1	2
Reconstruction revision	51	0	0	1	1	1	2
Augmentation/mastopexy revision	26	0	0	0	0	0	1
Augmentation/augmentation mastopexy	6	0	0	0	1	0	1
Mastopexy	10	1	0	0	0	0	0

Nine patients had a history of radiation and thin skin flaps resulting in a slower healing time and incorporation of the mesh implant. These patients were also found to have very thin skin flaps that required extra attention during the surgical procedure. Of the nine patients who had prior radiation, 44 % developed a postoperative complication. One primary reconstruction patient who had radiation after reconstruction had postoperative infection/extrusion. One secondary reconstruction patient had prior radiation and also experienced postoperative infection/extrusion. Two of the primary reconstruction patients who had prior radiation developed asymmetry that required an additional procedure. Some infection complications were noted early in the series and led to a new routine when placing the drains, using longer subcutaneous tunnels and a longer draining period.

## Discussion

Implant-based reconstruction is a very attractive option for women undergoing either therapeutic or prophylactic mastectomy. Those who benefit are patients who lack enough tissue required for autogenous reconstruction, those that have concerns related to donor site morbidities, patients concerned with scarring, and those that want to have little down-time postoperatively. Immediate implant-based reconstruction also has the particular benefit of giving the patient an instant positive psychological advantage. Stevens et al. [37] showed that patients who undergo immediate reconstruction compared to patients who have delayed reconstruction had a lower incidence of psychological morbidity postoperatively.

Immediate breast reconstruction with expanders has been performed using total muscle coverage of the prosthesis to protect the implant from exposure. It became apparent that the rigidity of the muscle often restricted inferior pole expansion resulting in a high-riding implant, an ill-defined inframammary fold, and less than desirable cosmetic results. With skin-sparing and nipple-sparing mastectomy gaining oncologic acceptance [15, 23], immediate implant-based reconstructions of the breast evolved, given the benefit of having a larger skin envelope. Despite the refinement in surgical techniques and the introduction of new prosthetic materials, reconstruction failure and implant dislocation are still common after prosthetic breast reconstruction.

The use of ADM to reinforce the muscle and to provide an increased area of coverage in the inferior pole of the breast led to promising improvements in these reconstructive challenges [9, 33, 35, 38]. Unfortunately, recent literature shows an increased rate of complications associated with the use of ADM in reconstructive breast procedures [10, 13, 17, 19, 21, 24, 40–43]. While this is controversial because there is literature suggesting a low complication profile with the use of ADMs, the experience of the senior author led him to look for alternatives to ADMs secondary to complications seen in his practice. Some of these complications include seromas, infections, and failure of vascularization. Brooke et al. [11] recently showed a total complication rate in all cases in which ADM was used of 17 % compared to 11 % in patients who did not receive ADM during reconstruction. Infectious complications were 10 % with ADM vs. 2 % without ADM. In a large retrospective analysis by Weichman et al. [42], of 628 immediate two-stage breast reconstructions, the use of ADM was associated with a significant increase in major complications. Also in question are the relative mechanical properties between individual sheets of allograft harvested from different donors. A study out of Harvard recently showed that there are statistically significant, highly variable elastic properties between sheets of ADM harvested from different donors [14]. This can be problematic when symmetry is crucial in breast reconstruction.

The use of ADM in corrective reconstructive breast surgery has also been reported in the literature [25, 26, 36]. Given the increasing evidence that ADM may be associated with increased complications, we aimed to examine another option that could prove to be a viable alternative to ADMs when used in select breast cases [1]. In this study we used the TIGR^®^ Matrix Surgical Mesh. The goal of the study was not to compare this product with ADMs or like products but to report on its use as temporary tissue reinforcement in a variety of plastic surgery breast cases.

TIGR^®^ Matrix is the first long-term resorbable synthetic mesh product. It is indicated for reinforcement of soft tissue that is weak and is manufactured from two different synthetic resorbable fibers. It has high strength during the first 6 months following implantation and is completely degraded and resorbed after ~3 years. TIGR^®^ Matrix is manufactured from the well-known and proven materials glycolide, lactide, and trimethylene carbonate, which degrade through hydrolysis and are cleared from the host tissue through normal metabolic pathways. We are now using it as an alternative to ADM in order to aid in correction of breast implant complications such as bottoming out, and as an adjunct in mastopexy surgery. The knitted structure of TIGR^®^ Matrix allows for easy handling and fixation while being strong and flexible. As shown in preclinical trials, it is rapidly vascularized, has a transient inflammatory response, and, over time, is replaced by well-organized connective tissue [18]. This was also our observation during this study. In patients who had a take-back procedure and thus whose mesh was biopsied, there was gross and histological evidence that the mesh was incorporating very nicely and being replaced by well-organized connective tissue. Since its market introduction in 2010, TIGR^®^ Matrix has been used in a variety of plastic and general surgical procedures where soft tissue reinforcement is required, and it has shown excellent results and performance with minimal complications [12].

We were pleased with the results seen in this patient series. The decision to use the matrix was based on the presence or absence of weakness or deficiency of tissue/pectoralis major muscle. In cases in which complete muscle coverage of a prosthesis was the goal, no mesh was used if complete muscle coverage was possible. It is not routine to use mesh in primary aesthetic procedures but it is beneficial at times in mastopexys to hold the tissue in position. The mesh was very easy to work with intraoperatively. The mesh is flexible and has excellent suture-holding ability. We have not encountered any significant restriction to expansion in those cases when the mesh was used. Very good aesthetic results were obtained in a variety of breast cases. Our total complication rate in patients requiring a revision was 15.2 %. The complication profile seems reasonable compared to that of ADMs. Specifically, postoperative seroma was seen in only 1.8 % of the total breasts reconstructed. The incidence of infections/extrusions was 3.6 %. These numbers are lower than those of many published studies referenced in this article. We have also proven (grossly and histologically) that this mesh incorporates very well into the patient’s native tissue. In order for a surgical mesh to be efficacious, it must allow vascular in-growth and incorporation into surrounding tissue. This mesh certainly meets these criteria.

Based on our results, a limitation of the matrix may be its use in severely radiated cases, as the complication rate was 44 % in patients who had radiation therapy. Complications were more common in radiated cases, with two infections/extrusions and two cases of asymmetry out of the nine patients who had radiation therapy. We now do all radiated-patient revisions in two stages, placing a Spectrum adjustable implant initially in reconstructive cases. Prolonged drainage was necessary in immediate reconstruction, often up to 2 weeks.

Infection complications were noted early in this series and led to a new routine when placing the drains, using longer subcutaneous tunnels and a longer draining period. These corrective actions reduced infections in the latter half of the patient series. Other complications seen included loss of inframammary fold and recurrent ptosis. These complications were seen in earlier cases where the mesh was not adequately positioned. Persistent rippling has also been seen in patients with very thin skin flaps. Overall, we were very pleased with the aesthetic results achieved in this patient series. As expected, there was a natural learning curve to using this new product, and as the study period went by the complication rate dropped off due to changes in technique.

In today’s healthcare environment, cost is becoming more important. A 10-cm × 15-cm sheet of TIGR^®^ Matrix costs $900. This is significantly less than the cost of ADMs and may be one potential advantage of using this matrix [16]. In our study, the cost of the mesh in cosmetic cases was absorbed into the total cost of the procedure.

This study is not without its limitations. First, this is a retrospective study. The sample size is relatively small. While our average follow-up is 16.5 months (longest was 26.1 months), we would like to follow these patients much longer to assess the durability of the postoperative results. A few key surgical techniques were also changed during the course of this study. However, we believe the change in techniques in the latter half of the study would likely improve the results. Also, there were no objective cosmetic assessments performed. However, that was not in the scope of this study. This study was not designed to be a comparative study between TIGR^®^ Matrix and ADMs or between patients receiving TIGR^®^ Matrix versus no mesh. Our goal was to prove that the TIGR^®^ Matrix could be an alternative to ADM when extra tissue support is needed in breast surgery cases.

The results of this study show that the synthetic long-term resorbable mesh TIGR^®^ Matrix Surgical Mesh can be used in patients undergoing implant-based breast reconstruction, breast surgery revisions, or cosmetic breast procedures, and the initial data reveal that it may be a viable alternative to acellular dermal matrices. As the product becomes more widely used and more data become available, we believe that the TIGR^®^ Matrix may have significant value for patients undergoing primary and secondary implant-based breast reconstruction as well as primary aesthetic procedures.

## Conclusion

The long-term absorbable synthetic matrix TIGR^®^ Matrix Surgical Mesh appears to have potential when used as temporary tissue reinforcement in patients undergoing breast reconstruction or breast surgery revision as well as primary aesthetic procedures, and it appears to be a possible alternative for acellular dermal matrices.
